# Rehabilitation with Poststroke Motor Recovery: A Review with a Focus on Neural Plasticity

**DOI:** 10.1155/2013/128641

**Published:** 2013-04-30

**Authors:** Naoyuki Takeuchi, Shin-Ichi Izumi

**Affiliations:** Department of Physical Medicine and Rehabilitation, Tohoku University Graduate School of Medicine, 2-1 Seiryo-cho, Aoba-ku, Sendai 980-8575, Japan

## Abstract

Motor recovery after stroke is related to neural plasticity, which involves developing new neuronal interconnections, acquiring new functions, and compensating for impairment. However, neural plasticity is impaired in the stroke-affected hemisphere. Therefore, it is important that motor recovery therapies facilitate neural plasticity to compensate for functional loss. Stroke rehabilitation programs should include meaningful, repetitive, intensive, and task-specific movement training in an enriched environment to promote neural plasticity and motor recovery. Various novel stroke rehabilitation techniques for motor recovery have been developed based on basic science and clinical studies of neural plasticity. However, the effectiveness of rehabilitative interventions among patients with stroke varies widely because the mechanisms underlying motor recovery are heterogeneous. Neurophysiological and neuroimaging studies have been developed to evaluate the heterogeneity of mechanisms underlying motor recovery for effective rehabilitation interventions after stroke. Here, we review novel stroke rehabilitation techniques associated with neural plasticity and discuss individualized strategies to identify appropriate therapeutic goals, prevent maladaptive plasticity, and maximize functional gain in patients with stroke.

## 1. Introduction

Despite advances in acute management, stroke remains a major cause of disability worldwide [1–6]. A number of neurological functions are impaired by stroke, the most common of which is motor disability contralateral to the stroke lesion side [7]. Therefore, many rehabilitation techniques based on motor learning paradigms have been developed to facilitate the recovery of impaired movement in patients with stroke [3, 8–11].

Neural plasticity can change central nervous system structure and/or function [12–15]. Recently, advances in technologies enabling noninvasive exploration of the human brain have increased our understanding of neural plasticity and its relationship to stroke recovery [9, 12, 16, 17]. Various novel stroke rehabilitative methods for motor recovery have been developed based on basic science and clinical studies characterizing brain remodeling due to neural plasticity [9, 11, 18]. The effectiveness of these approaches has been verified by systematic reviews and meta-analysis studies [8, 19–22]. However, responses to rehabilitative interventions show large inter-individual variation because the mechanisms underlying motor recovery are heterogeneous across patients [3, 8, 11, 23]. Furthermore, these mechanisms involve complex processes including restitution, substitution, and compensation that rely on a combination of spontaneous and learning-dependent processes [3, 24]. Therefore, elucidating the mechanisms underlying motor recovery can help to identify the most appropriate type, duration, and goals of individual rehabilitation strategies after stroke [11]. Neurophysiological and neuroimaging approaches have recently been developed to evaluate the heterogeneity of motor recovery mechanisms to better understand and predict the effectiveness of different rehabilitation interventions after stroke [12, 16, 25, 26].

In this review, we first discuss the principles of stroke rehabilitation in task-specific training and enriched environments. Then, we focus on novel strategies in stroke rehabilitation that are supported by evidence of associated neural plasticity. These approaches include constraint-induced movement therapy (CIMT), body weight-supported treadmill training (BWSTT), robotic training, transcutaneous neuromuscular electrical stimulation, noninvasive brain stimulation (NIBS), action observation, virtual reality (VR) training, and brain-computer interface (BCI). Finally, we discuss individualized strategies to inform the identification of therapeutic goals, to prevent maladaptive plasticity, and to maximize functional gain in patients with stroke.

## 2. Principles of Stroke Rehabilitation

Most protocols for stroke rehabilitation are based on motor learning, which induce dendrite sprouting, new synapse formation, alterations in existing synapses, and neurochemical production [10, 27]. These changes are thought to provide a mechanistic substrate to facilitate motor recovery after stroke [10, 27]. Motor learning is known to be greater if the practice method is meaningful, repetitive, and intensive [10, 17]. Further, it is recommended that stroke rehabilitation is applied in stroke care units where multidisciplinary teams can support active patient participation [9]. In this section, we review task-specific training and enriched environment therapeutic approaches that facilitate neural plasticity.

### 2.1. Task-Specific Training

Motor training after stroke should be targeted to goals that are relevant to the functional needs of the patient [10, 11]. Therefore, focusing on task-specific training to facilitate activities of daily living or other relevant motor tasks is a well-accepted principle of stroke rehabilitation [3]. This approach has been described by a variety of terms, including repetitive task practice, repetitive functional task practice, and task-oriented therapy [10, 28, 29]. Thus, task-specific training emphasizes the repetitive practice of skilled motor performance to improve individual functional abilities [10, 30]. Task-specific training can effectively recover a wide array of motor behaviors involving the upper limbs, lower limbs, sit-to-stand movements, and gait after stroke [29, 31–33]. Furthermore, repetitive task-specific training has been found to achieve better functional gains compared to nonrepetitive training [34, 35].

Increasing evidence suggests the involvement of neural plasticity in task-specific training [36, 37]. A meta-analysis of neurophysiological and neuroimaging studies has reported that neural changes in the sensorimotor cortex of the affected hemisphere accompany the gains in functional paretic upper extremity movements achieved with task-specific training [37]. Compared to traditional stroke rehabilitation approaches such as simple motor exercises, task-specific training induces long-lasting motor learning and associated cortical reorganization [30, 37]. Thus, there is strong evidence demonstrating that task-specific training can assist with functional motor recovery, which is driven by adaptive neural plasticity [8, 24, 30, 37, 38]. 

### 2.2. Enriched Environment

In addition to task specificity, the therapeutic environment plays an important role in stroke rehabilitation [39]. Environments that provide greater opportunity for physical activity and motivation are referred to as enriched environments [39]. Animal studies involving rat models of stroke have demonstrated that enriched environments facilitate motor recovery and neural plasticity because they present greater opportunities for physical activity, play, and social interactions compared to standard laboratory cages [39–41].

Clinically, stroke unit (SU) care administered by a well-coordinated multidisciplinary team can provide an enriched environment for patients with stroke [42]. SU care provides an organized package of care through a cyclical process involving the necessary elements of assessment, goal setting, intervention, and reassessment [3, 11]. Moreover, SU care provides individuals with a clear understanding of what is expected of them during task-specific training, resulting in neural plasticity that improves their performance [43]. Patient involvement in patient-centered interdisciplinary goal setting has been shown to encourage their motivation and engagement in therapy, resulting in better rehabilitation outcomes of impaired movement in patients with stroke [3]. Several studies have demonstrated that SU care had the greatest positive impact on disability levels after stroke [42, 44]. Moreover, the reported benefits of SU care extend to patients of all ages and to patients with varying stroke severity [44]. Thus, stroke rehabilitation programs should include meaningful, repetitive, intensive, and task-specific movement training in an enriched environment in order to promote neural plasticity and motor and functional recovery [10, 17].

## 3. Novel Strategies Based on Motor Training

During the last several decades, many studies have reported the use of novel motor learning-based stroke rehabilitation strategies [3, 8–11]. In this section focused on neural plasticity, we discuss several representative neurorehabilitation methods, including CIMT, BWSTT, and robot training.

### 3.1. CIMT

Patients with stroke often use the nonparetic limb instead of the paretic limb to perform daily activities. Dominant use of the nonparetic limb induces the phenomenon of learned nonuse in the paretic limb, which limits the capacity for subsequent gains in motor function [38, 45]. CIMT is a therapeutic strategy that was developed to overcome learned nonuse of the paretic limb. It forces paretic arm use by requiring a patient to perform functionally oriented activities while the nonparetic arm is physically restrained with a sling or glove. Mechanistically, the repetitive training of the paretic arm and constraint of the nonparetic upper arm used in CIMT might both be important for promoting neural plasticity. Skill acquisition with the nonparetic limb has been reported to negatively impact the use-dependent plasticity of the affected hemisphere in animal models of stroke [46]. The reasons underlying this constraint remain unclear, but this phenomenon may reflect use-dependent alterations in interhemispheric connectivity [47, 48]. Therefore, constraint of the nonparetic limb itself might ameliorate the impairment of use-dependent plasticity of the paretic limb after stroke [15, 45]. Several studies reported neural plasticity after CIMT as evidenced by neuroimaging and neurophysiological techniques [49–51]. Previous studies using transcranial magnetic stimulation (TMS) found that the cortical representation size of the paretic hand was increased after therapy [49, 50]. Neuroimaging studies also demonstrated altered neural network activity after CIMT [49, 51]. Moreover, a structural magnetic resonance imaging (MRI) study reported that CIMT increased gray matter in the bilateral sensorimotor cortices compared with control therapy [52]. Thus, there is evidence that CIMT induces both structural brain and physiological changes in patients with stroke [10].

 Wolf et al. conducted a multicenter single-blind randomized controlled trial known as the Extremity Constraint-Induced Therapy Evaluation Trial to compare the effects of 2-week CIMT with customary care in 222 individuals within 3–9 months of a first stroke [53]. At 1 year, the CIMT group performed better on functional tasks using the paretic upper limb. Moreover, the 2-year follow-up documented no decline from the 1-year assessment, and there were trends toward continued improvement of strength during the second year [54]. Most reviews of CIMT also report trends towards positive results of motor recovery in patients with chronic stroke [8–10]. However, previous studies had reported no significant differences in motor recovery between CIMT and an equal dose of traditional therapy for patients with acute stroke [55, 56]. This could be due to minimal or no learned nonuse during the acute phase [10]. Moreover, in the acute stage of stroke, high-intensity CIMT results in less improvement than low-intensity CIMT [56]. Therefore, additional studies are needed to explore optimal CIMT timing and intensity for motor recovery after stroke [11].

### 3.2. BWSTT

 BWSTT is a rehabilitation method in which patients with stroke walk on a treadmill with their body weight partially supported. BWSTT augments the ability to walk by enabling repetitive practice of complex gait cycles [57, 58]. In patients who have experienced a stroke, hemiparesis can cause abnormal control of the paretic lower limb, resulting in an asymmetrical gait pattern [59, 60]. Partial unloading of the lower extremities by the body weight support system results in straighter trunk and knee alignment during the loading phase of walking [61, 62]. BWSTT also improves swing time asymmetry, stride length, and walking speed [60, 62, 63]. Therefore, BWSTT allows the patient to practice nearly normal gait patterns and avoid developing compensatory walking habits, such as hip hiking and circumduction [58, 64].

There is evidence of gait improvement after BWSTT, including use of robotic device systems, compared to conventional therapy in patients with acute stroke and those with chronic stroke [60, 65, 66]. However, a recent study reported that the benefits of BWSTT were not superior to that achieved with home-based physical therapy that emphasized strength and balance, regardless of whether BWSTT was started 2 or 6 months after the stroke [67]. Moreover, among patients with severe walking impairments, multiple falls were more common in the group that received early BWSTT compared to the group that received late BWSTT and physical therapy [67]. Therefore, BWSTT programs should include balance training that helps prevent falls in patients, especially those with acute stroke and severe impairment. 

Mechanistically, BWSTT is believed to increase brain activity in the bilateral primary sensorimotor cortices, cingulate motor areas, caudate nuclei, and thalamus of the affected hemisphere [68]. Moreover, BWSTT has been found to alter central pattern generator activation in animal studies [69, 70]. In patients who have experienced a stroke, cerebral cortex function is impaired while that of the spinal cord is preserved. However, spinal cord changes may also be important for gait recovery after stroke due to changes in signals received following cerebral reorganization [71]. Thus, BWSTT can be used in patients with stroke to induce reorganization at the spinal and supraspinal levels, reduce gait parameter asymmetries, and increase walking speed. However, evidence of neural plasticity involved in this process is restricted to animal studies [71].

### 3.3. Robot Training

Robotic training offers several potential advantages in stroke rehabilitation, including good repeatability, precisely controllable assistance or resistance during movements, and objective and quantifiable measures of subject performance [72]. Moreover, robot training can provide the intensive and task-oriented type of training that has proven effective for promoting motor learning [8, 72]. These characteristics of robot training are thought to be useful for motor recovery after stroke. 

During the last decades, mechanically assisted robot training therapies have been developed for stroke rehabilitation to improve arm function [21, 73–75]. However, a multicenter, randomized controlled trial of patients with chronic stroke who had moderate-to-severe upper-limb impairment reported no difference in motor recovery between intensive physiotherapy and robot-assisted rehabilitative therapy [76]. Moreover, systematic reviews and meta-analyses have found no significant changes in activities of daily living ability after robotic training [77, 78]. Automated electromechanical gait machines have also been developed to facilitate lower limb rehabilitation. These machines consist of either a robot-driven exoskeleton orthosis or 2 electromechanical footplates that simulate gait phases [79–81]. Such machines are useful because they do not require therapists to set the paretic limbs and control weight shift, as is required for treadmill training [79, 80]. The use of electromechanical-assisted gait-training devices in combination with physiotherapy increases the chance of regaining independent walking ability after stroke but does not produce improvements in walking speed [82]. Therefore, in addition to automatic repetitive motor training, it is important for augmentation of robot training that robotic assistance is carried out in a minimum difference of input-output timing using electromyography (EMG) and/or position feedback [75, 83, 84]. Reducing these lag times is important because synchronization between sensory and motor information facilitates neural plasticity [85, 86]. Future studies are needed to determine the most appropriate characteristics of subjects and whether robot training has advantages over conventional therapy [75]. 

## 4. Augmentation of Use-Dependent Plasticity

Although the use-dependent plasticity induced by motor training is important for motor recovery after stroke, it has been reported that use-dependent plasticity is impaired in the affected hemisphere [87, 88]. Therefore, it is important to augment neural plasticity after stroke to facilitate motor recovery. In this section, we discuss the following possible methods of augmenting use-dependent plasticity in patients with stroke: transcutaneous neuromuscular electrical stimulation and NIBS.

### 4.1. Transcutaneous Neuromuscular Electrical Stimulation

Transcutaneous neuromuscular electrical stimulation can improve neuromuscular function in patients with stroke by strengthening muscles, increasing motor control, reducing spasticity, decreasing pain, and increasing range of motion [89]. Methods of transcutaneous neuromuscular electrical stimulation are generally categorized as either therapeutic electrical stimulation or functional electrical stimulation (FES). The defining feature of FES is that it provokes muscle contraction and produces a functionally useful movement during stimulation [89]. Several upper extremity FES devices are available, and the use of these devices seems to have a positive effect on upper-limb motor function in both acute and chronic stages of stroke [90–92]. FES has also been combined with different walking training strategies and has been shown to result in improvements in hemiplegic gait in both acute and chronic stages of stroke [93–95]. 

In addition to functional effects, FES is thought to have therapeutic effects, which are postulated to arise through the facilitation of neural plasticity by increasing the strength of afferent inputs [89]. In particular, FES supported by an EMG- or position-triggered system could induce appropriate proprioceptive feedback and promote motor learning [89, 96]. Patients can actively participate in intensive and repetitive task-specific training when they are responsible for initiating practice. Moreover, the synchronization of afferent feedback with voluntary movement by a biological signal-triggered system is useful for motor recovery because synchronization between the sensory and motor information facilitates neural plasticity [85, 86]. In fact, better performance is observed if paretic muscles are stimulated by voluntary muscular activity compared with nonsynchronized passive stimulation [97]. However, future research is needed to determine the most effective type and dose of electrical stimulation [98]. 

### 4.2. NIBS

Repetitive TMS (rTMS) and transcranial direct current stimulation (tDCS) are NIBS techniques that can alter human cortex excitability [99]. NIBS therapy for motor recovery following stroke aims to augment neural plasticity and improve motor function based on the interhemispheric competition model, which proposes that motor deficits in patients with stroke are due to reduced output from the affected hemisphere and excessive interhemispheric inhibition from the unaffected hemisphere to the affected hemisphere [18, 100, 101]. Therefore, NIBS achieves improvement in motor deficits by either increasing the excitability of the affected hemisphere or decreasing the excitability of the unaffected hemisphere [18, 102, 103]. Inhibitory NIBS increases excitability in the ipsilesional motor cortex by reducing excessive interhemispheric inhibition from the contralesional motor cortex [101, 104, 105]. Excitatory NIBS over the affected hemisphere directly increases the excitability of the ipsilesional motor cortex [105–108]. Motor cortex excitability enhancement appears to be required for motor learning [109, 110]. In fact, pairing of rehabilitative training with NIBS results in more enduring performance improvements and functional plasticity in the affected hemisphere compared with motor training or stimulation alone in patients with chronic stroke [101–104, 111]. Furthermore, cumulative NIBS has been shown to be important for continuous motor improvement in patients with stroke [112, 113]. This result indicates that neural plasticity is consolidated by cumulative NIBS intervention. Therefore, NIBS induces a more suitable environment for neural plasticity by artificially modulating the ipsilesional motor cortex, thus counteracting use-dependent plasticity impairment by facilitating plasticity in the affected hemisphere [18]. 

The effectiveness of NIBS is not limited to the chronic stage; it has been reported that both inhibitory and excitatory NIBS facilitate motor recovery in patients with stroke at the acute stage [108, 114–116]. However, another study reported that inhibitory and excitatory NIBS does not facilitate motor recovery in patients in acute stages of stroke [117, 118]. These discrepant findings underscore the importance of identifying the more effective type of NIBS, as well as optimal timing after stroke. A recent meta-analysis study of rTMS on upper-limb motor function in patients with stroke reported that inhibitory rTMS over the unaffected hemisphere might be more beneficial than excitatory rTMS over the affected hemisphere [22]. Although additional research has begun to evaluate the effectiveness of different NIBS stimulation protocols for motor recovery after stroke, further well-designed studies in larger populations are required to determine whether NIBS in the acute stroke stage can improve motor function and to identify the most effective NIBS protocols, including tDCS for stroke treatment [18].

## 5. Integration between Motor Learning and Multisensory Feedback

Multisensory feedback plays an important role in motor learning by reestablishing the sensorimotor loop that is disrupted by stroke [9]. Several multisensory feedback approaches have been reported for motor recovery in patients with stroke, including action observation and VR training [19, 119]. Recently developed BCI technology might also facilitate motor recovery by using robot devices and/or electrical stimulation [120].

### 5.1. Action Observation

There is increasing experimental evidence that some motor neural structures are recruited not only when actions are actually executed but also when the actions of another person are simply observed [121]. The neurophysiological basis for this recruitment is associated with mirror neurons, which have been identified in nonhuman primates [122, 123]. Human studies have also described a “mirror neuron system” involved in action understanding, imitation, motor learning, and modulating training effects [124–127]. According to the mirror neuron paradigm, action observation appears to activate the motor system similar to execution by generating an internal representation of action that can be targeted for motor learning [128–130]. A previous study in healthy subjects reported that observing another person learn a novel task improves subsequent performance of the same task [126]. Moreover, data from a recent virtual lesion study using TMS further supports the hypothesis that action observation coupled with physical practice may enhance use-dependent plasticity through the mirror neuron system in healthy controls [131].

Several clinical studies have reported that a combination of action observation therapy and physiotherapy improve upper-limb motor function in patients with chronic stroke [132, 133]. A recent multi-center randomized control trial demonstrated that action observation with physiotherapy has a positive effect on motor recovery in the acute stage of stroke [134]. Another study that employed functional MRI (fMRI) found that action observation facilitated motor recovery after stroke by reactivating the neural circuit containing the action observation/action execution matching system, which includes the bilateral ventral premotor cortex, supplementary motor area, and the contralateral supramarginal gyrus [132]. Therefore, increased activation of these areas suggests that the mirror neuron system (or its human homolog) may play an important role in motor learning and recovery related to action observation in patients with stroke [132, 134]. Moreover, action observation is safe and can be repetitively conducted without dependency on residual motor function. Despite the increasing evidence that action observation may become a useful strategy in stroke rehabilitation, future research is required to determine optimal practice intensity and duration before its translation into standard clinical practice [119].

### 5.2. VR

 VR is a computer-based technology that engages users in multisensory simulated environments, including real-time feedback (e.g., visual, auditory, and tactile feedback), allowing users to experience simulated real-world objects and events [135]. VR applications range from nonimmersive to fully immersive depending on the degree to which the user is isolated from the real surroundings when interacting with the virtual environment [136]. Immersive VR systems use large-screen projections, head-mounted displays, cave systems, or videocapture systems to immerse the user in a virtual environment [136]. In contrast, nonimmersive VR systems simply use a computer screen to simulate an experience with or without interface devices, such as a computer mouse, joystick, or force sensation [136]. VR exercise applications can easily provide patients with stroke with repetitive, intensive, and task-specific training and can apply relevant concepts for driving neural plasticity that produce motor function improvements after stroke [8, 136, 137]. Several studies have shown that the use of immersive VR results in practice-dependent enhancement of the affected arm by facilitating cortical reorganization [138, 139]. Moreover, a recent study has shown that video game applications that are classified as nonimmersive VR systems can be combined with conventional rehabilitation for upper arm improvement after stroke [140]. Video game systems have already been developed for home use, making this technology less costly and more accessible to clinicians and individuals [137]. Moreover, VR-based game systems can easily adjust task difficulty according to user capability [141, 142]. This encourages the user to train at optimal-level errors, inducing appropriate motivation and arousal, which are important for learning [143]. Therefore, VR-based game systems might be able to facilitate motor learning due to increased motivation of patients with stroke. However, the use of VR is not yet commonplace in clinical rehabilitation settings; only a few studies have been conducted, and the sample sizes were too small to draw firm conclusions [19].

### 5.3. BCI

BCI systems record, decode, and translate measurable neurophysiological signals into effector actions or behaviors without the use of peripheral physiological activities [144]. Several methods are available for detecting and measuring brain signals, including electroencephalography, electrocorticography, intracortical recordings, magnetoencephalography, fMRI, and functional near-infrared spectroscopy [144, 145]. One of the most popular neurophysiological phenomena assessed in BCI research is the modulation of sensorimotor rhythms through motor imagery [120, 144, 145]. The output of the BCI provides multisensory feedback to users, and this allows them to modulate their brain activity accordingly [144]. The feedback consists of sensory stimuli, such as visual, auditory, or tactile stimuli, and kinesthesia by robotic devices or FES [120, 145]. Therefore, BCI devices can couple intention with action and enable patients with stroke to achieve intended motor action [120, 144]. Considering that BCI technology is based on feedback and exploits learning mechanisms, BCI technology could be used to design and develop specific neurorehabilitation therapies for patients with stroke [120]. In fact, a recent study that combined motor training and motor imagery-based BCI reported a positive trend of upper-limb movement control in patients with stroke [146, 147]. BCI systems also might be useful for patients with severe stroke because they provide an alternative way of executing motor outputs through robotic devices [120, 144, 145]. Moreover, invasive BCI systems that utilize an intracortical recording technique have been developed in animal studies; these systems can detect signals, including synaptic and neuronal activities, and might facilitate neural plasticity due to accurate matching between motor intention and sensory feedback [145, 148–150]. However, the number of studies evaluating stroke recovery after BCI training is still limited. Future studies must evaluate the effect of BCI use on motor recovery after stroke and the role of BCI in neural plasticity [120]. 

## 6. Potential Individualized Rehabilitation Strategies for Appropriate Reorganization

An accurate prognosis of motor recovery after stroke can help to select individual rehabilitation strategies that promote appropriate reorganization [11]. It also is important for rehabilitation strategies to prevent maladaptive plasticity, which weakens motor function and limits motor recovery [15]. In this section, we discuss several potential individualized rehabilitation strategies to inform therapeutic goal setting, prevent maladaptive plasticity, and maximize functional gains in patients with stroke.

### 6.1. Imaging and Neurophysiological Findings Predict Motor Recovery

The simplest indicator of prognosis for patients with stroke is the degree of motor impairment. Many studies have suggested that motor outcomes are positively correlated with initial motor impairment after stroke [151–153]. However, the patterns of motor recovery are largely heterogeneous among patients with stroke; accurate prediction based on current motor impairment status alone can be difficult [11, 25]. Therefore, it has been suggested that motor recovery after stroke may be predicted more accurately using neurophysiological and neuroimaging findings [16, 25, 154]. Neurophysiological studies using TMS have revealed that ipsilesional corticospinal motor projection function is a good predictor of motor outcome after stroke [16, 25]. Neuroimaging studies using diffusion tensor imaging also have revealed that impairment of the ipsilesional corticospinal motor projections could predict motor recovery after stroke [25, 154]. Moreover, evaluation of the ipsilesional corticospinal tract function might facilitate the selection of rehabilitation strategies based on the prediction of potential functional gain, which is an individual's capacity for further functional improvement during the chronic stage of stroke recovery [25]. A recent study reported that the extent of injury to motor projections from supplementary and premotor areas of the affected hemisphere is also useful for predicting potential functional gains of paretic upper limbs from robot therapy in subjects with chronic stroke [26]. These results suggest that measures of motor tract function could be useful in estimating potential motor recovery of patients with stroke entering experimental neurorehabilitation trials and for patient selection in clinical trials [26]. Conversely, other studies have reported that the degree of ipsilesional corticospinal tract damage is not strongly associated with walking function [63, 155]. Moreover, the extent of lesion overlap with the corticospinal motor projections is only weakly correlated with therapy-related gains of gait function [63]. These findings support the importance of subcortical control, including the spinal cord, for lower-limb movements such as walking [156, 157]. Moreover, there is some evidence that ipsilateral motor projections are important in the recovery of walking function [158]. Therefore, the ipsilesional corticospinal motor projections appear to be less important in the control of walking than in the control of upper-limb dexterity after stroke [63].

In addition to motor tract function, identifying individual pattern of cortical activation may predict the effect of rehabilitation technique for patients with motor stroke. An fMRI study reported that lower baseline activity of the ipsilesional motor cortex during paretic hand movement was associated with greater functional gains after 6 weeks of rehabilitation therapy in chronic patients [159]. This result indicates that low baseline cortical activity might represent underuse of surviving cortical resources and possible responsiveness to rehabilitation therapy [159, 160]. Therefore, the motor projections may set a limit on the extent of recovery, but other parameters (e.g., preserved cortical activity) might be important when considering whether a patient has the capacity or potential to improve [160]. However, predicting functional gains by using individual cortical activity patterns may be more difficult than that by utilizing motor tract function. For example, a previous study reported that ipsilesional motor cortex excitability in good responders with chronic subcortical stroke for excitatory rTMS over the affected hemisphere is strongly activated, but not weakly, when moving the paretic hand before rTMS [161]. Moreover, functional gain has no direct correlation with ipsilesional motor cortex activity in the acute stage of stroke, but a pattern of cortical activation including the postcentral gyrus and cingulate cortex correlates with subsequent motor recovery [162]. Furthermore, in patients with stroke and severe initial hemiparesis, subsequent motor recovery was not predicted by task-related fMRI activation [163]. Thus, the effective neural activation pattern for neurorehabilitation might be different depending on time since stroke, lesion site, impairment of motor function, and/or rehabilitation technique due to the heterogeneous mechanisms underlying motor recovery and neurorehabilitation techniques.

Genetic factors of neural plasticity-related components should also be considered to affect the capacity of an individual patient's brain to recover motor function [164, 165]. Moreover, genetic variation might be able to explain some of the variability encountered in motor rehabilitation efficacy [23, 165, 166]. It has been reported that various genetic factors influence neural plasticity in animals and humans (for review see [165]). However, there is no evidence regarding individualized rehabilitation strategies using genetic information.

### 6.2. Preventing Maladaptive Plasticity

Although some neural plasticity undoubtedly contributes to motor recovery after stroke, it remains unclear whether all forms of neural plasticity contribute to genuine motor recovery [12, 14, 167]. Maladaptive plasticity that weakens motor function and limits recovery has recently been reported after stroke [15, 46, 48, 100, 168]. Therefore, it is important for individual stroke rehabilitation strategies to prevent maladaptive plasticity.

Several studies have suggested that neural plasticity associated with compensatory movement might contribute to maladaptive plasticity after stroke [15, 38]. To perform daily tasks, patients with stroke often develop a compensatory hyperreliance on the nonparetic side, proximal paretic side, or trunk movement [169–172]. However, this strong and efficient motor compensation may prevent the affected side from generating normal motor patterns for daily activities [38, 169]. In particular, dominant use of the nonparetic limb induces learned nonuse of the paretic limb and limits its functional improvement [38, 45]. The facilitation of neural plasticity underlying compensatory learning with the nonparetic limb after stroke also exacerbates use-dependent plasticity impairment of the affected hemisphere via abnormal interhemispheric inhibition [47, 48]. CIMT that combines a rehabilitative training regime for the paretic limb with constraint of the nonparetic limb can overcome learned nonuse of the paretic limb and has been shown to improve motor function in patients with stroke [45, 53, 173]. Therefore, clinicians should consider CIMT for patients having stroke who fit its criteria to facilitate appropriate reorganization and prevent maladaptive plasticity. However, patients with stroke and severe motor function impairments are not suitable candidates for CIMT therapy. Studies of animal stroke models suggest that compensatory use of the nonparetic limb while the paretic limb is being used does not necessarily result in learned nonuse [46]. Therefore, patients with stroke and poor motor function who engage in compensatory use of the nonparetic limb in daily activities may benefit from bilateral movement training to prevent learned nonuse of the paretic side [15, 25].

Increased activity of the paretic proximal arm due to compensatory movement may contribute to the abnormal interjoint movement in the proximal limb that is, often observed after a stroke [172]. Therefore, the selected rehabilitation program may have to avoid intense training of the paretic proximal side. To our knowledge, no rehabilitation program currently addresses this problem, and compensatory movement of the paretic proximal muscle is useful for reaching in some patients with stroke and poor motor function [38, 174]. Thus, at least in cases where patients with stroke have good motor function, a rehabilitation program that avoids compensatory use of the paretic proximal side may be helpful.

## 7. Conclusion

Most stroke rehabilitation protocols are based on motor learning to induce neural plasticity, which refers to the ability of the brain to develop new neuronal interconnections, acquire new functions, and compensate for impairment. These changes are greater if the practice method is meaningful, repetitive, and intensive. It is recommended that rehabilitation take place in stroke care units that can provide an organized package of care through a cyclical process involving assessment, goal setting, intervention, and reassessment. Systematic reviews and meta-analyses have verified the effects of developed techniques in stroke rehabilitation. CIMT that combines a rehabilitative training regime for the paretic limb with constraint of the nonparetic limb can overcome learned nonuse of the paretic limb and has been shown to improve motor function in patients with stroke. BWSTT may induce reorganization at the spinal and supraspinal levels by providing normal gait programs, reducing asymmetries of gait parameters, and increasing walking speed. Robotic training can provide repetitive motor training and reduce the therapists' physical load. Transcutaneous neuromuscular electrical stimulation and NIBS can improve motor recovery by ameliorating use-dependent plasticity impairment after stroke. Moreover, novel stroke rehabilitation strategies such as action observation, VR, and BCI have been developed based on multisensory feedback, which plays an important role in learning to control human brain signals and in re-establishing the sensorimotor loop disrupted by stroke.

Current clinical practice for stroke rehabilitation is based on accumulating evidence from neural plasticity studies. However, responses to rehabilitative interventions show large interindividual variation due to the heterogeneity of mechanisms underlying motor recovery. Therefore, an accurate prediction of motor recovery can help to determine the type, duration, and goals for individual stroke rehabilitation strategies. An assessment of corticospinal integrity using neurophysiological and imaging techniques might be useful for predicting motor recovery and setting individualized rehabilitation goals. However, numerous other factors influence behavioral responses to therapy, including injury to other brain structures, psychosocial factors, and age. Moreover, it is important for appropriate reorganization after stroke to prevent maladaptive plasticity, which weakens motor function and limits motor recovery.

Early stroke rehabilitation is critical for enhancing motor recovery, but the optimal time window for specific neurorehabilitation has yet to be elucidated. The intensity and duration of the rehabilitation strategy are also important factors that influence effectiveness. Although the evidence base for stroke rehabilitation continues to grow, future studies must be conducted to ascertain the optimal time, intensity, and duration for specific rehabilitation techniques and to facilitate the translation of basic scientific evidence into routine clinical application.

## References

[1] Kolominsky-Rabas PL, Weber M, Gefeller O, Neundoerfer B, Heuschmann PU (2001). Epidemiology of ischemic stroke subtypes according to TOAST criteria: incidence, recurrence, and long-term survival in ischemic stroke subtypes: a population-based study. *Stroke*.

[2] Kwakkel G, Kollen BJ, Wagenaar RC (2002). Long term effects of intensity of upper and lower limb training after stroke: a randomised trial. *Journal of Neurology Neurosurgery and Psychiatry*.

[3] Langhorne P, Bernhardt J, Kwakkel G (2011). Stroke rehabilitation. *The Lancet*.

[4] Balami JS, Buchan AM (2012). Complications of intracerebral haemorrhage. *The Lancet Neurology*.

[5] Taqi MA, Vora N, Callison RC, Lin R, Wolfe TJ (2012). Past, present, and future of endovascular stroke therapies. *Neurology*.

[6] Chollet F, Albucher JF (2012). Strategies to augment recovery after stroke. *Current Treatment Options in Neurology*.

[7] Hankey GJ, Jamrozik K, Broadhurst RJ, Forbes S, Anderson CS (2002). Long-term disability after first-ever stroke and related prognostic factors in the Perth Community Stroke Study, 1989-1990. *Stroke*.

[8] Langhorne P, Coupar F, Pollock A (2009). Motor recovery after stroke: a systematic review. *The Lancet Neurology*.

[9] Johansson BB (2011). Current trends in stroke rehabilitation. A review with focus on brain plasticity. *Acta Neurologica Scandinavica*.

[10] Arya KN, Pandian S, Verma R, Garg RK (2011). Movement therapy induced neural reorganization and motor recovery in stroke: a review. *Journal of Bodywork and Movement Therapies*.

[11] Brewer L, Horgan F, Hickey A, Williams D (2013). Stroke rehabilitation: recent advances and future therapies. *QJM*.

[12] Chen H, Epstein J, Stern E (2010). Neural plasticity after acquired brain injury: evidence from functional neuroimaging. *PM & R*.

[13] Hosp JA, Luft AR (2011). Cortical plasticity during motor learning and recovery after ischemic stroke. *Neural Plasticity*.

[14] Dancause N, Nudo RJ (2011). Shaping plasticity to enhance recovery after injury. *Progress in Brain Research*.

[15] Takeuchi N, Izumi S (2012). Maladaptive plasticity for motor recovery after stroke: mechanisms and approaches. *Neural Plasticity*.

[16] Talelli P, Greenwood RJ, Rothwell JC (2006). Arm function after stroke: neurophysiological correlates and recovery mechanisms assessed by transcranial magnetic stimulation. *Clinical Neurophysiology*.

[17] Kleim JA, Jones TA (2008). Principles of experience-dependent neural plasticity: implications for rehabilitation after brain damage. *Journal of Speech, Language, and Hearing Research*.

[18] Takeuchi N, Izumi S (2012). Noninvasive brain stimulation for motor recovery after stroke: mechanisms and future views. *Stroke Research and Treatment*.

[19] Laver KE, George S, Thomas S, Deutsch JE, Crotty M (2011). Virtual reality for stroke rehabilitation. *Cochrane Database of Systematic Reviews*.

[20] Nijland R, Kwakkel G, Bakers J, van Wegen E (2011). Constraint-induced movement therapy for the upper paretic limb in acute or sub-acute stroke: a systematic review. *International Journal of Stroke*.

[21] Mehrholz J, Hadrich A, Platz T, Kugler J, Pohl M (2012). Electromechanical and robot-assisted arm training for improving generic activities of daily living, arm function, and arm muscle strength after stroke. *Cochrane Database of Systematic Reviews*.

[22] Hsu WY, Cheng CH, Liao KK, Lee IH, Lin YY (2012). Effects of repetitive transcranial magnetic stimulation on motor functions in patients with stroke: a meta-analysis. *Stroke*.

[23] Floel A, Cohen LG (2010). Recovery of function in humans: cortical stimulation and pharmacological treatments after stroke. *Neurobiology of Disease*.

[24] Kwakkel G, Kollen B, Lindeman E (2004). Understanding the pattern of functional recovery after stroke: facts and theories. *Restorative Neurology and Neuroscience*.

[25] Stinear CM, Barber PA, Smale PR, Coxon JP, Fleming MK, Byblow WD (2007). Functional potential in chronic stroke patients depends on corticospinal tract integrity. *Brain*.

[26] Riley JD, Le V, Der-Yeghiaian L (2011). Anatomy of stroke injury predicts gains from therapy. *Stroke*.

[27] Mulder T, Hochstenbach J (2001). Adaptability and flexibility of the human motor system: implications for neurological rehabilitation. *Neural Plasticity*.

[28] French B, Thomas LH, Leathley MJ (2007). Repetitive task training for improving functional ability after stroke. *Cochrane Database of Systematic Reviews*.

[29] Hubbard IJ, Parsons MW, Neilson C, Carey LM (2009). Task-specific training: evidence for and translation to clinical practice. *Occupational Therapy International*.

[30] Bayona NA, Bitensky J, Salter K, Teasell R (2005). The role of task-specific training in rehabilitation therapies. *Topics in Stroke Rehabilitation*.

[31] Monger C, Carr JH, Fowler V (2002). Evaluation of a home-based: exercise and training programme to improve sit-to-stand in patients with chronic stroke. *Clinical Rehabilitation*.

[32] Peurala SH, Pitkänen K, Sivenius J, Tarkka IM (2004). How much exercise does the enhanced gait-oriented physiotherapy provide for chronic stroke patients?. *Journal of Neurology*.

[33] Arya KN, Verma R, Garg RK, Sharma VP, Agarwal M, Aggarwal GG (2012). Meaningful task-specific training (MTST) for stroke rehabilitation: a randomized controlled trial. *Topics in Stroke Rehabilitation*.

[34] Salbach NM, Mayo NE, Wood-Dauphinee S, Hanley JA, Richards CL, Côté R (2004). A task-orientated intervention enhances walking distance and speed in the first year post stroke: a randomized controlled trial. *Clinical Rehabilitation*.

[35] Michaelsen SM, Dannenbaum R, Levin MF (2006). Task-specific training with trunk restraint on arm recovery in stroke: randomized control trial. *Stroke*.

[36] Jang SH, Kim YH, Cho SH, Lee JH, Park JW, Kwon YH (2003). Cortical reorganization induced by task-oriented training in chronic hemiplegic stroke patients. *NeuroReport*.

[37] Richards LG, Stewart KC, Woodbury ML, Senesac C, Cauraugh JH (2008). Movement-dependent stroke recovery: a systematic review and meta-analysis of TMS and fMRI evidence. *Neuropsychologia*.

[38] Levin MF, Kleim JA, Wolf SL (2009). What do motor “recovery” and “compensationg” mean in patients following stroke?. *Neurorehabilitation and Neural Repair*.

[39] Döbrössy MD, Dunnett SB (2001). The influence of environment and experience on neural grafts. *Nature Reviews Neuroscience*.

[40] Biernaskie J, Corbett D (2001). Enriched rehabilitative training promotes improved forelimb motor function and enhanced dendritic growth after focal ischemic injury. *The Journal of Neuroscience*.

[41] Nithianantharajah J, Hannan AJ (2006). Enriched environments, experience-dependent plasticity and disorders of the nervous system. *Nature Reviews Neuroscience*.

[42] Stroke Unit Trialists' Collaboration (2007). Organised inpatient (stroke unit) care for stroke. *Cochrane Database of Systematic Reviews*.

[43] Davis JZ (2006). Task selection and enriched environments: a functional upper extremity training program for stroke survivors. *Topics in Stroke Rehabilitation*.

[44] Saposnik G, Kapral MK, Coutts SB, Fang J, Demchuk AM, Hill MD (2009). Do all age groups benefit from organized inpatient stroke care?. *Stroke*.

[45] Taub E, Uswatte G, Mark VW, Morris DM (2006). The learned nonuse phenomenon: implications for rehabilitation. *Europa Medicophysica*.

[46] Allred RP, Jones TA (2008). Maladaptive effects of learning with the less-affected forelimb after focal cortical infarcts in rats. *Experimental Neurology*.

[47] Allred RP, Cappellini CH, Jones TA (2010). TThe, “good” limb makes the, “bad” limb worse: experience-dependent interhemispheric disruption of functional outcome after cortical infarcts in rats. *Behavioral Neuroscience*.

[48] Kerr AL, Cheng SY, Jones TA (2011). Experience-dependent neural plasticity in the adult damaged brain. *Journal of Communication Disorders*.

[49] Wittenberg GF, Chen R, Ishii K (2003). Constraint-induced therapy in stroke: magnetic-stimulation motor maps and cerebral activation. *Neurorehabilitation and Neural Repair*.

[50] Liepert J, Haevernick K, Weiller C, Barzel A (2006). The surround inhibition determines therapy-induced cortical reorganization. *NeuroImage*.

[51] Schaechter JD, Kraft E, Hilliard TS (2002). Motor recovery and cortical reorganization after constraint-induced movement therapy in stroke patients: a preliminary study. *Neurorehabilitation and Neural Repair*.

[52] Gauthier LV, Taub E, Perkins C, Ortmann M, Mark VW, Uswatte G (2008). Remodeling the brain: plastic structural brain changes produced by different motor therapies after stroke. *Stroke*.

[53] Wolf SL, Winstein CJ, Miller JP (2006). Effect of constraint-induced movement therapy on upper extremity function 3 to 9 months after stroke: the EXCITE randomized clinical trial. *Journal of the American Medical Association*.

[54] Wolf SL, Winstein CJ, Miller JP (2008). Retention of upper limb function in stroke survivors who have received constraint-induced movement therapy: the EXCITE randomised trial. *The Lancet Neurology*.

[55] Boake C, Noser EA, Ro T (2007). Constraint-induced movement therapy during early stroke rehabilitation. *Neurorehabilitation and Neural Repair*.

[56] Dromerick AW, Lang CE, Birkenmeier RL (2009). Very early constraint-induced movement during stroke rehabilitation (VECTORS): a single-center RCT. *Neurology*.

[57] Hesse S (2004). Recovery of gait and other motor functions after stroke: novel physical and pharmacological treatment strategies. *Restorative Neurology and Neuroscience*.

[58] Ifejika-Jones NL, Barrett AM (2011). Rehabilitation—emerging technologies, innovative therapies, and future objectives. *Neurotherapeutics*.

[59] von Schroeder HP, Coutts RD, Lyden PD, Billings E, Nickel VL (1995). Gait parameters following stroke: a practical assessment. *Journal of Rehabilitation Research and Development*.

[60] Laufer Y, Dickstein R, Chefez Y, Marcovitz E (2001). The effect of treadmill training on the ambulation of stroke survivors in the early stages of rehabilitation: a randomized study. *Journal of Rehabilitation Research and Development*.

[61] Visintin M, Barbeau H (1989). The effects of body weight support on the locomotor pattern of spastic paretic patients. *Canadian Journal of Neurological Sciences*.

[62] Lindquist ARR, Prado CL, Barros RML, Mattioli R, da Costa PHL, Salvini TF (2007). Gait training combining partial body-weight support, a treadmill, and functional electrical stimulation: effects on poststroke gait. *Physical Therapy*.

[63] Dawes H, Enzinger C, Johansen-Berg H (2008). Walking performance and its recovery in chronic stroke in relation to extent of lesion overlap with the descending motor tract. *Experimental Brain Research*.

[64] Chen G, Patten C (2006). Treadmill training with harness support: selection of parameters for individuals with poststroke hemiparesis. *Journal of Rehabilitation Research and Development*.

[65] Barbeau H, Visintin M (2003). Optimal outcomes obtained with body-weight support combined with treadmill training in stroke subjects. *Archives of Physical Medicine and Rehabilitation*.

[66] Mayr A, Kofler M, Quirbach E, Matzak H, Fröhlich K, Saltuari L (2007). Prospective, blinded, randomized crossover study of gait rehabilitation in stroke patients using the Lokomat gait orthosis. *Neurorehabilitation and Neural Repair*.

[67] Duncan PW, Sullivan KJ, Behrman AL (2011). Body-weight—supported treadmill rehabilitation after stroke. *The New England Journal of Medicine*.

[68] Enzinger C, Dawes H, Johansen-Berg H (2009). Brain activity changes associated with treadmill training: after stroke. *Stroke*.

[69] MacKay-Lyons M (2002). Central pattern generation of locomotion: a review of the evidence. *Physical Therapy*.

[70] Norton JA, Mushahwar VK (2010). Afferent inputs to mid- and lower-lumbar spinal segments are necessary for stepping in spinal cats. *Annals of the New York Academy of Sciences*.

[71] Verma R, Arya KN, Sharma P, Garg RK (2012). Understanding gait control in post-stroke: implications for management. *Journal of Bodywork and Movement Therapies*.

[72] Belda-Lois JM, Mena-del Horno S, Bermejo-Bosch I (2011). Rehabilitation of gait after stroke: a review towards a top-down approach. *Journal of Neuroengineering and Rehabilitation*.

[73] Hesse S, Werner C, Pohl M, Rueckriem S, Mehrholz J, Lingnau ML (2005). Computerized arm training improves the motor control of the severely affected arm after stroke: a single-blinded randomized trial in two centers. *Stroke*.

[74] Masiero S, Celia A, Rosati G, Armani M (2007). Robotic-assisted rehabilitation of the upper limb after acute stroke. *Archives of Physical Medicine and Rehabilitation*.

[75] Lum PS, Godfrey SB, Brokaw EB, Holley RJ, Nichols D (2012). Robotic approaches for rehabilitation of hand function after stroke. *American Journal of Physical Medicine and Rehabilitation*.

[76] Lo AC, Guarino PD, Richards LG (2010). Robot-assisted therapy for long-term upper-limb impairment after stroke. *The New England Journal of Medicine*.

[77] Prange GB, Jannink MJA, Groothuis-Oudshoorn CGM, Hermens HJ, Ijzerman MJ (2006). Systematic review of the effect of robot-aided therapy on recovery of the hemiparetic arm after stroke. *Journal of Rehabilitation Research and Development*.

[78] Kwakkel G, Kollen BJ, Krebs HI (2008). Effects of robot-assisted therapy on upper limb recovery after stroke: a systematic review. *Neurorehabilitation and Neural Repair*.

[79] Hesse S, Schmidt H, Werner C, Bardeleben A (2003). Upper and lower extremity robotic devices for rehabilitation and for studying motor control. *Current Opinion in Neurology*.

[80] Banala SK, Kim SH, Agrawal SK, Scholz JP (2009). Robot assisted gait training with active leg exoskeleton (ALEX). *IEEE Transactions on Neural Systems and Rehabilitation Engineering*.

[81] Pinter MM, Brainin M (2012). Rehabilitation after stroke in older people. *Maturitas*.

[82] Mehrholz J, Werner C, Kugler J, Pohl M (2007). Electromechanical-assisted training for walking after stroke. *Cochrane Database of Systematic Reviews*.

[83] Stein J, Narendran K, McBean J, Krebs K, Hughes R (2007). Electromyography-controlled exoskeletal upper-limb-powered orthosis for exercise training after stroke. *American Journal of Physical Medicine and Rehabilitation*.

[84] Hu XL, Tong KY, Song R, Zheng XJ, Leung WWF (2009). A comparison between electromyography-driven robot and passive motion device on wrist rehabilitation for chronic stroke. *Neurorehabilitation and Neural Repair*.

[85] Stefan K, Kunesch E, Cohen LG, Benecke R, Classen J (2000). Induction of plasticity in the human motor cortex by paired associative stimulation. *Brain*.

[86] Wolters A, Sandbrink F, Schlottmann A (2003). A temporally asymmetric Hebbian rule governing plasticity in the human motor cortex. *Journal of Neurophysiology*.

[87] Carmichael ST (2006). Cellular and molecular mechanisms of neural repair after stroke: making waves. *Annals of Neurology*.

[88] Di Filippo M, Tozzi A, Costa C (2008). Plasticity and repair in the post-ischemic brain. *Neuropharmacology*.

[89] Schuhfried O, Crevenna R, Fialka-Moser V, Paternostro-Sluga T (2012). Non-invasive neuromuscular electrical stimulation in patients with central nervous system lesions: an educational review. *Journal of Rehabilitation Medicine*.

[90] Alon G, Levitt AF, McCarthy PA (2007). Functional electrical stimulation enhancement of upper extremity functional recovery during stroke rehabilitation: a pilot study. *Neurorehabilitation and Neural Repair*.

[91] Knutson JS, Harley MY, Hisel TZ, Hogan SD, Maloney MM, Chae J (2012). Contralaterally controlled functional electrical stimulation for upper extremity hemiplegia: an early-phase randomized clinical trial in subacute stroke patients. *Neurorehabilitation and Neural Repair*.

[92] Alon G, McBride K, Ring H (2002). Improving selected hand functions using a noninvasive neuroprosthesis in persons with chronic stroke. *Journal of Stroke and Cerebrovascular Diseases*.

[93] Kottink AIR, Oostendorp LJM, Buurke JH, Nene AV, Hermens HJ, IJzerman MJ (2004). The orthotic effect of functional electrical stimulation on the improvement of walking in stroke patients with a dropped foot: a systematic review. *Artificial Organs*.

[94] Yan T, Hui-Chan CWY, Li LSW (2005). Functional electrical stimulation improves motor recovery of the lower extremity and walking ability of subjects with first acute stroke: a randomized placebo-controlled trial. *Stroke*.

[95] Laufer Y, Ring H, Sprecher E, Hausdorff JM (2009). Gait in individuals with chronic hemiparesis: one-year follow-up of the effects of a neuroprosthesis that ameliorates foot drop. *Journal of Neurologic Physical Therapy*.

[96] Chae J (2003). Neuromuscular electrical stimulation for motor relearning in hemiparesis. *Physical Medicine and Rehabilitation Clinics of North America*.

[97] Barsi GI, Popovic DB, Tarkka IM, Sinkjær T, Grey MJ (2008). Cortical excitability changes following grasping exercise augmented with electrical stimulation. *Experimental Brain Research*.

[98] Pomeroy VM, King L, Pollock A, Baily-Hallam A, Langhorne P (2006). Electrostimulation for promoting recovery of movement or functional ability after stroke. *Cochrane Database of Systematic Reviews*.

[99] Lefaucheur JP (2009). Methods of therapeutic cortical stimulation. *Neurophysiologie Clinique*.

[100] Murase N, Duque J, Mazzocchio R, Cohen LG (2004). Influence of interhemispheric interactions on motor function in chronic stroke. *Annals of Neurology*.

[101] Takeuchi N, Chuma T, Matsuo Y, Watanabe I, Ikoma K (2005). Repetitive transcranial magnetic stimulation of contralesional primary motor cortex improves hand function after stroke. *Stroke*.

[102] Nowak DA, Grefkes C, Ameli M, Fink GR (2009). Interhemispheric competition after stroke: brain stimulation to enhance recovery of function of the affected hand. *Neurorehabilitation and Neural Repair*.

[103] Takeuchi N, Tada T, Toshima M, Matsuo Y, Ikoma K (2009). Repetitive transcranial magnetic stimulation over bilateral hemispheres enhances motor function and training effect of paretic hand in patients after stroke. *Journal of Rehabilitation Medicine*.

[104] Takeuchi N, Tada T, Toshima M, Chuma T, Matsuo Y, Ikoma K (2008). Inhibition of the unaffected motor cortex by 1 HZ repetitive transcranial magnetic stimulation enhances motor performance and training effect of the paretic hand in patients with chronic stroke. *Journal of Rehabilitation Medicine*.

[105] Fregni F, Boggio PS, Mansur CG (2005). Transcranial direct current stimulation of the unaffected hemisphere in stroke patients. *NeuroReport*.

[106] Hummel F, Celnik P, Giraux P (2005). Effects of non-invasive cortical stimulation on skilled motor function in chronic stroke. *Brain*.

[107] Kim YH, You SH, Ko MH (2006). Repetitive transcranial magnetic stimulation-induced corticomotor excitability and associated motor skill acquisition in chronic stroke. *Stroke*.

[108] Di Lazzaro V, Profice P, Pilato F (2010). Motor cortex plasticity predicts recovery in acute stroke. *Cerebral Cortex*.

[109] Pascual-Leone A, Tarazona F, Keenan J, Tormos JM, Hamilton R, Catala MD (1998). Transcranial magnetic stimulation and neuroplasticity. *Neuropsychologia*.

[110] Muellbacher W, Zlemann U, Wissel J (2002). Early consolidation in human primary motor cortex. *Nature*.

[111] Zimerman M, Heise KF, Hoppe J, Cohen LG, Gerloff C, Hummel FC (2012). Modulation of training by single-session transcranial direct current stimulation to the intact motor cortex enhances motor skill acquisition of the paretic hand. *Stroke*.

[112] Fregni F, Boggio PS, Valle AC (2006). A sham-controlled trial of a 5-day course of repetitive transcranial magnetic stimulation of the unaffected hemisphere in stroke patients. *Stroke*.

[113] Boggio PS, Nunes A, Rigonatti SP, Nitsche MA, Pascual-Leone A, Fregni F (2007). Repeated sessions of noninvasive brain DC stimulation is associated with motor function improvement in stroke patients. *Restorative Neurology and Neuroscience*.

[114] Liepert J, Zittel S, Weiller C (2007). Improvement of dexterity by single session low-frequency repetitive transcranial magnetic stimulation over the contralesional motor cortex in acute stroke: a double-blind placebo-controlled crossover trial. *Restorative Neurology and Neuroscience*.

[115] Khedr EM, Abdel-Fadeil MR, Farghali A, Qaid M (2009). Role of 1 and 3 Hz repetitive transcranial magnetic stimulation on motor function recovery after acute ischaemic stroke. *European Journal of Neurology*.

[116] Khedr EM, Etraby AE, Hemeda M, Nasef AM, Razek AAE (2010). Long-term effect of repetitive transcranial magnetic stimulation on motor function recovery after acute ischemic stroke. *Acta Neurologica Scandinavica*.

[117] Seniow J, Bilik M, Lesniak M, Waldowski K, Iwanski S, Czlonkowska A (2012). Transcranial magnetic stimulation combined with physiotherapy in rehabilitation of poststroke hemiparesis: a randomized, double-blind, placebo-controlled study. *Neurorehabilitation and Neural Repair*.

[118] Rossi C, Sallustio F, Di Legge S, Stanzione P, Koch G (2013). Transcranial direct current stimulation of the affected hemisphere does not accelerate recovery of acute stroke patients. *European Journal of Neurology*.

[119] Sale P, Franceschini M (2012). Action observation and mirror neuron network: a tool for motor stroke rehabilitation. *European Journal of Physical and Rehabilitation Medicine*.

[120] Silvoni S, Ramos-Murguialday A, Cavinato M (2011). Brain-computer interface in stroke: a review of progress. *Clinical EEG and Neuroscience*.

[121] Jeannerod M (2001). Neural simulation of action: a unifying mechanism for motor cognition. *NeuroImage*.

[122] Rizzolatti G, Fadiga L, Gallese V, Fogassi L (1996). Premotor cortex and the recognition of motor actions. *Cognitive Brain Research*.

[123] Gallese V, Fadiga L, Fogassi L, Rizzolatti G (1996). Action recognition in the premotor cortex. *Brain*.

[124] Iacoboni M, Woods RP, Brass M, Bekkering H, Mazziotta JC, Rizzolatti G (1999). Cortical mechanisms of human imitation. *Science*.

[125] Flanagan JR, Johansson RS (2003). Action plans used in action observation. *Nature*.

[126] Mattar AAG, Gribble PL (2005). Motor learning by observing. *Neuron*.

[127] Celnik P, Stefan K, Hummel F, Duque J, Classen J, Cohen LG (2006). Encoding a motor memory in the older adult by action observation. *NeuroImage*.

[128] Ortigue S, Sinigaglia C, Rizzolatti G, Grafton ST (2010). Understanding actions of others: the electrodynamics of the left and right hemispheres. A high-density EEG neuroimaging study. *PLoS ONE*.

[129] Rizzolatti G, Craighero L (2004). The mirror-neuron system. *Annual Review of Neuroscience*.

[130] Rizzolatti G, Sinigaglia C (2010). The functional role of the parieto-frontal mirror circuit: interpretations and misinterpretations. *Nature Reviews Neuroscience*.

[131] Cantarero G, Galea JM, Ajagbe L, Salas R, Willis J, Celnik P (2011). Disrupting the ventral premotor cortex interferes with the contribution of action observation to use-dependent plasticity. *Journal of Cognitive Neuroscience*.

[132] Ertelt D, Small S, Solodkin A (2007). Action observation has a positive impact on rehabilitation of motor deficits after stroke. *NeuroImage*.

[133] Celnik P, Webster B, Glasser DM, Cohen LG (2008). Effects of action observation on physical training after stroke. *Stroke*.

[134] Franceschini M, Ceravolo MG, Agosti M (2012). Clinical relevance of action observation in upper-limb stroke rehabilitation: a possible role in recovery of functional dexterity. A randomized clinical trial. *Neurorehabilitation and Neural Repair*.

[135] Weiss P, Kizony R, Feintuch U, Katz N, Selzer M, Cohen L, Gage F, Clarke S, Duncan P (2006). Virtual reality in neurorehabilitation. *Textbook of Neural Repair and Rehabilitation*.

[136] Henderson A, Korner-Bitensky N, Levin M (2007). Virtual reality in stroke rehabilitation: a systematic review of its effectiveness for upper limb motor recovery. *Topics in Stroke Rehabilitation*.

[137] Saposnik G, Levin M (2011). Virtual reality in stroke rehabilitation: a meta-analysis and implications for clinicians. *Stroke*.

[138] Jang SH, You SH, Hallett M (2005). Cortical reorganization and associated functional motor recovery after virtual reality in patients with chronic stroke: an experimenter-blind preliminary study. *Archives of Physical Medicine and Rehabilitation*.

[139] You SH, Jang SH, Kim YH (2005). Virtual reality-induced cortical reorganization and associated locomotor recovery in chronic stroke: an experimenter-blind randomized study. *Stroke*.

[140] Saposnik G, Teasell R, Mamdani M (2010). Effectiveness of virtual reality using wii gaming technology in stroke rehabilitation: a pilot randomized clinical trial and proof of principle. *Stroke*.

[141] Cameirão MS, Badia SBI, Oller ED, Verschure PFMJ (2010). Neurorehabilitation using the virtual reality based rehabilitation gaming system: methodology, design, psychometrics, usability and validation. *Journal of NeuroEngineering and Rehabilitation*.

[142] Cameirao MD, Bermudez IBS, Duarte E, Verschure PF (2011). Virtual reality based rehabilitation speeds up functional recovery of the upper extremities after stroke: a randomized controlled pilot study in the acute phase of stroke using the rehabilitation gaming system. *Restorative Neurology and Neuroscience*.

[143] Green CS, Bavelier D (2008). Exercising your brain: a review of human brain plasticity and training-induced learning. *Psychology and Aging*.

[144] Birbaumer N, Murguialday AR, Cohen L (2008). Brain-computer interface in paralysis. *Current Opinion in Neurology*.

[145] Daly JJ, Wolpaw JR (2008). Brain-computer interfaces in neurological rehabilitation. *The Lancet Neurology*.

[146] Ang KK, Guan C, Chua KS (2009). A clinical study of motor imagery-based brain-computer interface for upper limb robotic rehabilitation. *Conference Proceedings of the Annual International Conference of the IEEE Engineering in Medicine and Biology Society*.

[147] Prasad G, Herman P, Coyle D, McDonough S, Crosbie J (2010). Applying a brain-computer interface to support motor imagery practice in people with stroke for upper limb recovery: a feasibility study. *Journal of NeuroEngineering and Rehabilitation*.

[148] Hatsopoulos NG, Donoghue JP (2009). The science of neural interface systems. *Annual Review of Neuroscience*.

[149] Andersen RA, Hwang EJ, Mulliken GH (2010). Cognitive neural prosthetics. *Annual Review of Psychology*.

[150] Lebedev MA, Nicolelis MA (2011). Toward a whole-body neuroprosthetic. *Progress in Brain Research*.

[151] Smania N, Paolucci S, Tinazzi M (2007). Active finger extension: a simple movement predicting recovery of arm function in patients with acute stroke. *Stroke*.

[152] Beebe JA, Lang CE (2009). Active range of motion predicts upper extremity function 3 months after stroke. *Stroke*.

[153] Nijland RHM, van Wegen EEH, Harmeling-van der Wel BC, Kwakkel G (2010). Presence of finger extension and shoulder abduction within 72 hours after stroke predicts functional recovery: early prediction of functional outcome after stroke: the EPOS cohort study. *Stroke*.

[154] Jang SH (2011). A review of diffusion tensor imaging studies on motor recovery mechanisms in stroke patients. *NeuroRehabilitation*.

[155] Ahn YH, Ahn SH, Kim H, Hong JH, Jang SH (2006). Can stroke patients walk after complete lateral corticospinal tract injury of the affected hemisphere?. *NeuroReport*.

[156] Dimitrijevic MR, Gerasimenko Y, Pinter MM (1998). Evidence for a spinal central pattern generator in humans. *Annals of the New York Academy of Sciences*.

[157] Hultborn H, Nielsen JB (2007). Spinal control of locomotion—from cat to man. *Acta Physiologica*.

[158] Jang SH, You SH, Kwon YH, Hallett M, Mi YL, Sang HA (2005). Cortical reorganization associated lower extremity motor recovery as evidenced by functional MRI and diffusion tensor tractography in a stroke patient. *Restorative Neurology and Neuroscience*.

[159] Cramer SC, Parrish TB, Levy RM (2007). Predicting functional gains in a stroke trial. *Stroke*.

[160] Ward N (2011). Assessment of cortical reorganisation for hand function after stroke. *The Journal of Physiology*.

[161] Ameli M, Grefkes C, Kemper F (2009). Differential effects of high-frequency repetitive transcranial magnetic stimulation over ipsilesional primary motor cortex in cortical and subcortical middle cerebral artery stroke. *Annals of Neurology*.

[162] Marshall RS, Zarahn E, Alon L, Minzer B, Lazar RM, Krakauer JW (2009). Early imaging correlates of subsequent motor recovery after stroke. *Annals of Neurology*.

[163] Zarahn E, Alon L, Ryan SL (2011). Prediction of motor recovery using initial impairment and fMRI 48 h poststroke. *Cerebral Cortex*.

[164] Stinear C (2010). Prediction of recovery of motor function after stroke. *The Lancet Neurology*.

[165] Pearson-Fuhrhop KM, Cramer SC (2010). Genetic influences on neural plasticity. *PM & R*.

[166] Cramer SC (2008). A window into the molecular basis of human brain plasticity. *The Journal of Physiology*.

[167] Johnston MV (2009). Plasticity in the developing brain: implications for rehabilitation. *Developmental Disabilities Research Reviews*.

[168] Takeuchi N, Tada T, Chuma T, Matsuo Y, Ikoma K (2007). Disinhibition of the premotor cortex contributes to a maladaptive change in the affected hand after stroke. *Stroke*.

[169] Roby-Brami A, Feydy A, Combeaud M, Biryukova EV, Bussel B, Levin MF (2003). Motor compensation and recovery for reaching in stroke patients. *Acta Neurologica Scandinavica*.

[170] Dobkin BH (2005). Rehabilitation after stroke. *The New England Journal of Medicine*.

[171] Schallert T (2006). Behavioral tests for preclinical intervention assessment. *NeuroRx*.

[172] Schwerin S, Dewald JPA, Haztl M, Jovanovich S, Nickeas M, MacKinnon C (2008). Ipsilateral versus contralateral cortical motor projections to a shoulder adductor in chronic hemiparetic stroke: implications for the expression of arm synergies. *Experimental Brain Research*.

[173] Liepert J, Bauder H, Miltner WHR, Taub E, Weiller C (2000). Treatment-induced cortical reorganization after stroke in humans. *Stroke*.

[174] Roby-Brami A, Jacobs S, Bennis N, Levin MF (2003). Hand orientation for grasping and arm joint rotation patterns in healthy subjects and hemiparetic stroke patients. *Brain Research*.

